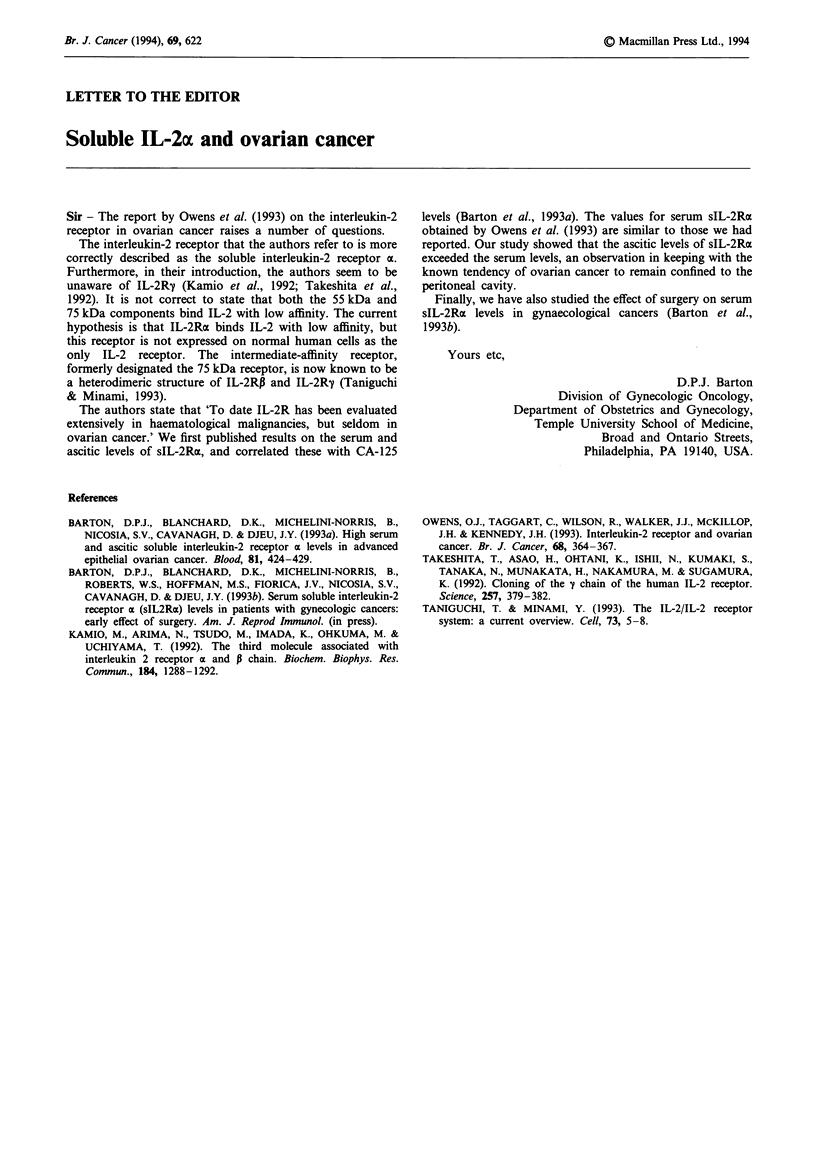# Soluble IL-2 alpha and ovarian cancer.

**DOI:** 10.1038/bjc.1994.117

**Published:** 1994-03

**Authors:** D. P. Barton


 © Macmillan Press Ltd., 1994

LETTER TO THE EDITOR

### Soluble IL-2α and ovarian cancer

**Sir** – The report by Owens *et al.* (1993) on the interleukin-2 receptor in ovarian cancer raises a number of questions.

The interleukin-2 receptor that the authors refer to is more correctly described as the soluble interleukin-2 receptor α. Furthermore, in their introduction, the authors seem to be unaware of IL-2Rγ (Kamio *et al.*, 1992; Takeshita *et al.*, 1992). It is not correct to state that both the 55 kDa and 75 kDa components bind IL-2 with low affinity. The current hypothesis is that IL-2Rα binds IL-2 with low affinity, but this receptor is not expressed on normal human cells as the only IL-2 receptor. The intermediate-affinity receptor, formerly designated the 75 kDa receptor, is now known to be a heterodimeric structure of IL-2Rβ and IL-2Rγ (Taniguchi & Minami, 1993).

The authors state that 'To date IL-2R has been evaluated extensively in haematological malignancies, but seldom in ovarian cancer.' We first published results on the serum and ascitic levels of sIL-2Rα, and correlated these with CA-125 levels (Barton *et al.*, 1993*a*). The values for serum sIL-2Rα obtained by Owens *et al.* (1993) are similar to those we had reported. Our study showed that the ascitic levels of sIL-2Rα exceeded the serum levels, an observation in keeping with the known tendency of ovarian cancer to remain confined to the peritoneal cavity.

Finally, we have also studied the effect of surgery on serum sIL-2Rα levels in gynaecological cancers (Barton *et al.*, 1993*b*).

Yours etc,

D.P.J. Barton
Division of Gynecologic Oncology,
Department of Obstetrics and Gynecology,
Temple University School of Medicine,
Broad and Ontario Streets,
Philadelphia, PA 19140, USA.

### References

BARTON, D.P.J., BLANCHARD, D.K., MICHELINI-NORRIS, B., NICOSIA, S.V., CAVANAGH, D. & DJEU, J.Y. (1993*a*). High serum and ascitic soluble interleukin-2 receptor α levels in advanced epithelial ovarian cancer. *Blood*, **81**, 424–429.

BARTON, D.P.J., BLANCHARD, D.K., MICHELINI-NORRIS, B., ROBERTS, W.S., HOFFMAN, M.S., FIORICA, J.V., NICOSIA, S.V., CAVANAGH, D. & DJEU, J.Y. (1993*b*). Serum soluble interleukin-2 receptor α (sIL2Rα) levels in patients with gynecologic cancers: early effect of surgery. *Am. J. Reprod Immunol.* (in press).

KAMIO, M., ARIMA, N., TSUDO, M., IMADA, K., OHKUMA, M. & UCHIYAMA, T. (1992). The third molecule associated with interleukin 2 receptor α and β chain. *Biochem. Biophys. Res. Commun.*, **184**, 1288–1292.

OWENS, O.J., TAGGART, C., WILSON, R., WALKER, J.J., McKILLOP, J.H. & KENNEDY, J.H. (1993). Interleukin-2 receptor and ovarian cancer. *Br. J. Cancer*, **68**, 364–367.

TAKESHITA, T., ASAO, H., OHTANI, K., ISHII, N., KUMAKI, S., TANAKA, N., MUNAKATA, H., NAKAMURA, M. & SUGAMURA, K. (1992). Cloning of the γ chain of the human IL-2 receptor. *Science*, **257**, 379–382.

TANIGUCHI, T. & MINAMI, Y. (1993). The IL-2/IL-2 receptor system: a current overview. *Cell*, **73**, 5–8.